# Dinutuximab Beta Versus Naxitamab in the Treatment of Relapsed/Refractory Neuroblastoma in Patients with Stable Disease, Minor Response or Partial Response and Disease in Bone or Bone Marrow: Systematic Review and Matching-Adjusted Indirect Comparison

**DOI:** 10.3390/cancers17172723

**Published:** 2025-08-22

**Authors:** Holger N. Lode, Przemysław Holko, Aleksandra Wieczorek, Nikolai Siebert, Dominique Valteau-Couanet, Alberto Garaventa, Adela Cañete, John Anderson, Isaac Yaniv, Shifra Ash, Juliet Gray, Roberto Luksch, Carla Manzitti, Sascha Troschke-Meurer, Torsten Ebeling, Paweł Kawalec, Katarzyna Śladowska, Ruth L. Ladenstein

**Affiliations:** 1 Department of Pediatric Hematology and Oncology, University Medicine Greifswald, 17489 Greifswald, Germany; nikolai.siebert@med.uni-greifswald.de (N.S.); sascha.troschke-meurer@med.uni-greifswald.de (S.T.-M.); torsten.ebeling@med.uni-greifswald.de (T.E.); 2 Department of Nutrition and Drug Research, Institute of Public Health, Faculty of Health Sciences, Jagiellonian University Medical College, 31-008 Krakow, Polandpawel.kawalec@uj.edu.pl (P.K.); katarzyna.wojcieszek@uj.edu.pl (K.Ś.); 3 Paediatric Haematology Oncology, Jagiellonian University Medical College, 31-008 Krakow, Poland; a.wieczorek@uj.edu.pl; 4 Children and Adolescent Oncology Department, Gustave Roussy, Paris-Sud University, 94805 Paris, France; dominiqu.valteau@gustaveroussy.fr; 5 Oncology Unit, IRCCS Istituto Giannina Gaslini, 16147 Genoa, Italy; alberto.garaventa@gaslini.org (A.G.); carlamanzitti@gaslini.org (C.M.); 6 Hospital Universitario y Politecnico La Fe, 46026 Valencia, Spain; adela.canete@uv.es; 7 UCL Great Ormond Street Institute of Child Health, London WC1N 1EH, UK; j.anderson@ucl.ac.uk; 8 Schneider Children’s Medical Center of Israel, Sackler Faculty of Medicine Tel Aviv University, Petach Tikva 4920235, Israel; iyaniv@clalit.org.il; 9 Department of Pediatric Hematology-Oncology, Ruth Rappaport Children’s Hospital, Rambam Health Care Campus, Technion-Israel Institute of Technology, Rappaport Faculty of Medicine, Haifa 3109601, Israel; s_ash@rambam.health.gov.il; 10 Centre for Cancer Immunology, University of Southampton, Southampton SO16 6YD, UK; juliet.gray@uhs.nhs.uk; 11 Fondazione IRCCS Istituto Nazionale dei Tumori, 20133 Milan, Italy; roberto.luksch@institutotumori.mi.it; 12 Department of Paediatrics, St. Anna Children‘s Hospital, Medical University, 1090 Vienna, Austria

**Keywords:** relapsed/refractory neuroblastoma, dinutuximab beta, naxitamab, matching-adjusted indirect comparison, systematic review

## Abstract

**Objective:** Dinutuximab beta (DB) and naxitamab (NAXI) with GM-CSF are used for maintenance treatment of relapsed/refractory neuroblastoma. The objective of this study was to systematically assess comparative efficacy of the two therapies within their designated indications in accordance with established clinical guidelines. **Methods:** Relevant evidence was identified in systematic literature review. Individual patient data (IPD) from prospective clinical trials of DB were assessed and data on patients with disease in bone or bone marrow, as assessed in MRI, CT, mIBG or biopsy, with incomplete response to previous therapy were included. Patients with complete response, progressive disease and/or soft tissue disease were excluded. DB population was adjusted for sex, MYCN amplification, disease type (relapsed, refractory), and disease site (bone marrow and/or bone) to balance aggregated characteristics of NAXI population. More characteristics were included in sensitivity analyses, including DB treatment without interleukin-2, as currently recommended. Overall response rate (ORR) was assessed as best response. **Results:** Aggregated data for NAXI from Study 201 (*n* = 52) and Study 230 (*n* = 38) and IPD from DB studies (APN311-202, APN311-304, *c* = 77) met the inclusion criteria. Compared to NAXI, DB significantly extended progression-free survival (PFS): hazard ratio, DB vs. NAXI of 0.47 (95% CI: 0.26 to 0.87, *p* = 0.015). ORR was 60.1% (95% CI: 48.5% to 71.6%) for DB vs. 43.3% (33.1% to 53.6%) for NAXI (ORR odds ratio, DB vs. NAXI was 1.97, 95% CI: 1.02 to 3.80, *p* = 0.044). Sensitivity analyses and unadjusted comparisons supported the results. **Conclusion:** In the indirect comparison, dinutuximab beta significantly extended PFS and increased ORR compared to naxitamab.

## Simple Summary

No studies directly comparing dinutuximab beta (DB) and naxitamab (NAXI) in the maintenance treatment of relapsed/refractory neuroblastoma were identified in a systematic literature review. An indirect comparison was conducted based on the results of independent studies of DB and NAXI. Individual patient data from DB studies were adjusted to ensure that the characteristics of the patients were similar in the groups treated with the two antibodies. The study demonstrated that DB resulted in a longer survival period without the deterioration of neuroblastoma (referred to as progression-free survival) in comparison to NAXI (*p* = 0.015). In addition, a higher proportion of patients exhibited a positive response to DB in comparison to NAXI therapy (*p* = 0.044).

## 1. Introduction

Neuroblastoma, the most common solid extracranial tumour in children, remains a major challenge in paediatric oncology. Despite the introduction of new treatment strategies, including high-dose chemotherapy followed by autologous bone marrow (BM) or stem cell transplantation (SCT), clinical trial results [1,2] show that the outcomes of patients with high-risk neuroblastoma remain poor, secondary to relapses occurring even after extensive multimodal interventions [3]. The development of effective adjuvant therapeutic strategies was identified as the only viable approach for further enhancing outcomes in this disease, with tumour-specific immunotherapy developed to target both newly diagnosed and relapsed disease [4,5,6].

A disialoganglioside (GD2), highly expressed on the surface of neuroblastoma cells, is a suitable target for immunotherapy [5,6,7]. The introduction of anti-GD2 antibodies was a breakthrough in the treatment of high-risk neuroblastoma (HR-NBL). Maintenance therapy based on anti-GD2 antibodies, such as dinutuximab and dinutuximab beta (DB), has proved to be effective for patients with HR-NBL in the first-line setting [8]; COG and SIOPEN studies show an approximate 15% improvement in survival benefit (event-free survival and overall survival) (*p* < 0.001) but no additional benefit when adding a cytokine (interleukin-2 [IL-2]) [9]. This led to the recommendation to use anti-GD2 without IL-2. In Europe, DB is currently the only anti-GD2 antibody approved by the European Medicines Agency (EMA) since May 2017 for the treatment of neuroblastoma. It is a chimeric monoclonal IgG1 antibody produced in Chinese hamster ovary (CHO) cells [10]. DB is indicated both in first-line maintenance and in relapsed/refractory settings [10]. In 2020, a humanised anti-GD2 antibody was registered for relapsed/refractory neuroblastoma by the Food and Drug Administration (FDA)—naxitamab (NAXI), previously known as hu3F8 [11]. Both DB and NAXI can bind to GD2 on the cell surface and induce complement-dependent cytotoxicity (CDC) and antibody-dependent cell-mediated cytotoxicity (ADCC) [11,12,13,14]. While NAXI was shown to have greater affinity to GD2 than DB [15], stronger binding does not necessarily translate into greater efficacy [16]. Indeed, DB was shown to mediate stronger ADCC effector function when used in equimolar amounts [14]. Granulocyte–macrophage colony-stimulating factor (GM-CSF) is co-administered with NAXI to enhance its cytotoxic activity [11]; however, comparative prospective clinical evidence supporting the added benefit of this cytokine is lacking. Comparative analyses indicated that infusions of DB (in combination with 13-cis-retinoic acid and IL-2) significantly extended overall survival in patients with relapsed/refractory neuroblastoma, compared to patients who did not receive immunotherapy (HR = 0.52 and 0.60 versus different historical controls) [17]. NAXI was approved by the FDA under accelerated approval based on the overall response rate (34–45%) and duration of response (median, 6.2 months) from two single-arm, ongoing studies: 12-201 and 230 [11]. Based on randomised controlled trial results, anti-GD2 immunotherapies have also become the standard of care in relapsed/refractory neuroblastoma in combination with chemotherapy [18,19]. Anti-GD2 monotherapy is subsequently provided as maintenance depending on disease control [20,21,22]. There are no head-to-head trials comparing the anti-GD2 immunotherapies DB and NAXI in the treatment of relapsed/refractory neuroblastoma. Nonetheless, these two interventions can be compared indirectly utilising aggregated, published results from NAXI trials and individual patient data (IPD) from DB trials.

The licenced indication of DB [10] is broader than that of NAXI [11] because it also includes maintenance therapy in newly diagnosed HR-NBL and in patients with both partial and complete response to prior therapy. In relapsed/refractory settings, DB is registered for maintenance in patients with or without residual disease, irrespective of its site, stabilised by other appropriate measures, but without specific response criteria [10]. NAXI is registered only for patients with relapsed or refractory HR-NBL in the bone or bone marrow who have demonstrated a partial response, a minor response or stable disease following prior therapy [11]. This narrower indication of NAXI was the focus of this review. Therefore, our objective was to indirectly compare the efficacy of DB and NAXI in the treatment of relapsed/refractory neuroblastoma in patients with stable disease, minor response or partial response, and disease in bone or bone marrow. We also sought to compare the treatments in the subgroup of patients treated with DB without IL-2, as currently recommended. Harmonised inclusion criteria were applied to the DB populations (to reflect the inclusion criteria of the NAXI clinical trials), and matching-adjusted indirect comparison (MAIC) was chosen to balance the patient characteristics in the two populations.

## 2. Materials and Methods

### 2.1. Data Sources and Inclusion Criteria

A systematic review of the literature was performed according to the recommendations of the Preferred Reporting Items for Systematic reviews and Meta-Analyses (PRISMA) [23] and the Cochrane Handbook [24] to identify clinical studies that could be included in the indirect comparison of DB (±IL-2) and NAXI (+GM-CSF) for the treatment of relapsed or refractory neuroblastoma in bone or bone marrow in patients who had demonstrated a partial response, minor response or stable disease following previous therapy.

The search was conducted using MEDLINE (via PubMed), EMBASE and the Cochrane Library databases in April 2025. The search strategy was based on the MeSH terms combined with Boolean logical operators. The reference lists of the included studies and the websites of the EMA, FDA, ESMO, ASCO, ISPOR, and others with study results were also searched (the strategies are described in detail in Supplementary Tables S1–S8). A study was included if it met prespecified criteria: (1) patient population: aged 12 months and above with relapsed, refractory and/or recurrent neuroblastoma; (2) treatment: assessed interventions including DB used in maintenance therapy, in combination with IL-2 or as single agent (as recommended by SIOPEN) at registered dosage, or NAXI + GM-CSF with dosing according to FDA prescribing information (patients with complete response or progressive disease before use of this drug were excluded, according to the approved indication) [11]; (3) study type: randomised controlled trials (RCTs) and non-randomised studies, including control, observational, single-arm studies with ≥10 participants. Clinical study reports, full text articles, data from registration documents, in the absence of any references published as full texts, and abstracts (posters and conference presentations) with very recent results were allowed. The outcomes of interest were as follows: event free survival (EFS), progression free survival (PFS), overall survival (OS), and/or overall response rate (ORR; defined as complete [CR] or partial response [PR]) (Supplementary Tables S9 and S10). Editorials, letters, data from clinical trial registers, reviews, and studies with DB or NAXI used with chemotherapy combinations (chemoimmunotherapy) were excluded.

Studies were selected according to the PRISMA recommendations [23]. The titles and abstracts of the studies identified during the search were screened, and a list of studies that met the inclusion criteria was generated. The next step was to select studies based on full-version articles, considering all the inclusion and exclusion criteria for the analysis. Studies were selected by two independent reviewers (K.Ś. and P.K.), and any disagreements at any stage were resolved by discussion, consultation with a third reviewer (A.W.), and finally by consensus. However, there was a high degree of compatibility among the reviewers (99%).

### 2.2. Study Quality Assessment and Data Extraction

The quality of eligible RCTs was evaluated using the Cochrane risk-of-bias tool 2.0 for randomised trials [24,25]. This tool allows the assessment of the following domains: allocation sequence generation, allocation concealment, deviations from intended interventions, missing outcome data, outcome measurement, selective reporting, and “other issues”. The domain-based assessment allows the following ratings to be assigned to each domain: low risk of bias (“+”), high risk of bias (“−”) or some concerns (“?”). The overall risk score was based on the highest level of risk identified in one of these domains. The NICE scale was used for single-arm studies (case series). Data from the included studies were extracted independently by 2 reviewers (K.Ś. and P.H.) using a predefined data extraction form. The following information was extracted and analysed to assess the homogeneity of the studies: design (methodology), key inclusion/exclusion criteria, treatment regimen, and availability of data for outcomes of interest.

### 2.3. Data Analysis and Synthesis

After assessing the homogeneity of the studies and the availability of IPD, the possibility of conducting an unanchored MAIC was evaluated. The baseline characteristics of the participants in the included studies and the outcomes of interest were extracted. The study selection criteria for the MAIC were as follows: (i) trial design: prospective studies; (ii) patient population: studies with expected target population sizes of at least 10 patients (target population: those with relapsed or refractory neuroblastoma in bone or bone marrow but not in soft tissues, who had demonstrated a partial response, a minor response or stable disease following previous therapy); (iii) treatment: according to the registered schedule and/or clinical guidelines.

MAIC was carried out according to Signorovitch et al. [26,27] and the NICE DSU Technical Support Document [28]. Harmonised inclusion criteria were applied to DB populations (to reflect the inclusion criteria of NAXI clinical trials, that is, relapsed or refractory neuroblastoma in bone or bone marrow in patients who had demonstrated a partial response, a minor response or stable disease following previous therapy; the absence of soft tissue disease), and MAIC was performed to balance the populations regarding key baseline patient characteristics. MAIC weighting was based on the estimated propensity to enrol in the DB trials vs. NAXI trials. No information on predictors of the effectiveness of NAXI or DB among patients in the target population was available. In the pivotal NAXI trial, subgroup analysis was performed only for ORR [29]. Consequently, MAIC was performed for patient characteristics that changed the ORR compared to the entire sample in [30] by more than 10%. The other criteria for including patient characteristics in the MAIC were (i) availability (limited information on the characteristics of the patients enrolled in NAXI trials), and (ii) the number of patients with a specific characteristic among the patients enrolled in DB studies (the characteristics that described fewer than 5 patients in the sample were excluded from the base case analysis, which prevented assigning excessive weight to a patient). In the base-case analysis, 5 patient characteristics were included in the adjustment process: MYCN amplification, refractory disease, female sex and disease site described by two variables (bone marrow only; bone and bone marrow). Other characteristics (prior radiotherapy, Black race, missing MYCN, missing International Neuroblastoma Staging System [INSS], and stage 3 according to the INSS) that have no proven impact on efficacy (prior radiotherapy) or those which described 1 or 2 patients only in the DB dataset were included in the sensitivity analyses.

Sensitivity analyses were performed for (i) DB monotherapy (no IL-2 treatment, as the impact of its addition on treatment benefits is not supported by evidence); (ii) the selection of MAIC characteristics (the addition of characteristics with unknown impacts on the results and/or that affected low numbers of patients); and (iii) selection of the NAXI studies (without trial 230).

MAIC weights were considered as sampling weights in Kaplan–Meier analyses, logistic regression models and Cox regression models. All the hazard ratios (HRs) and odds ratios (ORs) with 95% confidence intervals (CIs) are presented for DB compared to NAXI. The χ^2^ test was applied to compare patient characteristics between groups. A logistic regression model (with or without MAIC weights) with the grouping variable only (DB vs. NAXI) was used to assess the differences in ORR between groups.

A *p*-value of less than 0.05 was considered significant. Data were prepared and analysed using StataNow 19.5SE (StataCorp., College Station, TX, USA); OriginPro 2025 (OriginLab Corporation, Northampton, MA, USA); and R: A language and environment for statistical computing (R Foundation for Statistical Computing, Vienna, Austria).

## 3. Results

### 3.1. Search Results and Included Studies

The database search identified 521 records in medical databases (Supplementary Figure S1). After full-text review, 10 studies were included in the review. These included three pivotal studies for NAXI: 12-201 (NCT03363373) [29,30,31,32]; 12-230 (NCT01757626) [32]; and 2PR01—compassionate use [32]. They also included six pivotal studies for DB: Wieczorek et al., 2023 [33]; APN311-304 [34,35]; Flaadt et al., 2023 [36]; Mueller et al., 2018 [37]/APN311-303 [17]; APN311-202 stage I (V1+V2 cohorts) and stage II (V3 cohort) [17,38]; and APN311-101 (Ladenstein et al., 2013) [39]. Among the identified studies, only one RCT was found—APN311-202 stage II (V3 cohort) [17,38], which had a high risk of bias due to its open-label design. The remaining studies were single-arm, with moderate to high quality according to the NICE scale. The full list of references for the identified studies is provided in the Supplementary Material; information on the methodology and quality assessment of the studies is provided in Supplementary Tables S11–S15.

### 3.2. Study Selection for Indirect Comparison

Four studies were included in the MAIC: Study 201 (aggregated data on the ORR and patient characteristics; individual patient data on PFS and OS) [30] and Study 230 (aggregated data on the ORR and patient characteristics) [32] for NAXI and IPD from DB trials: APN311-304 [35] and APN311-202 (cohorts V1+V2 and cohort V3) [38]. Only Study 201 could be included in the analysis of PFS, while both Study 201 and 203 had data available for the analysis of ORR. Consequently, the populations for the MAIC of the two outcomes were different. Other studies were excluded from the MAIC based on the predefined criteria (Table 1).

Information from 52 patients treated with NAXI in Study 201 (efficacy cohort; data cut-off: 31 December 2021) was used [30]. Individual patient data on the PFS, ORR and OS from Study 201 were obtained from the published study supplement [30]. Information from Study 230 that enrolled patients from the target population for NAXI treatment was only available from the FDA document (*n* = 38; group 1 + 3 only; data on ORR only) [32].

**Figure 1:**
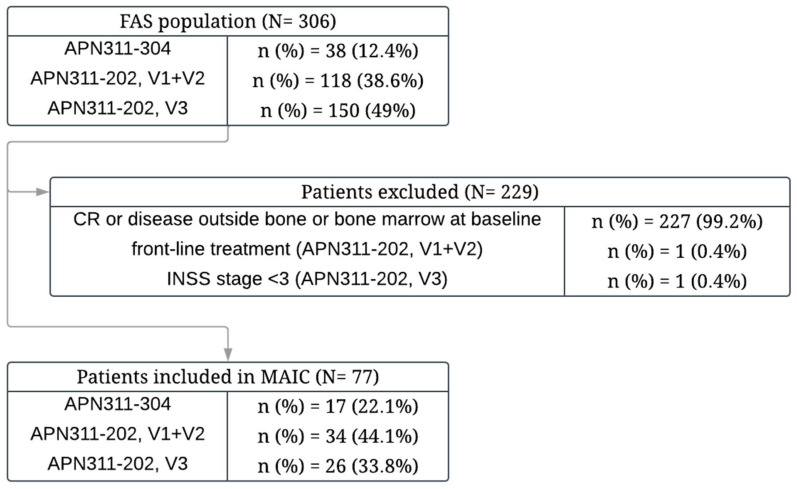
Patient flow diagram. CR, complete response; FAS, full analysis set; MAIC, matched-adjusted indirect comparison.

The IPD from the APN311-304 trial and two phases of the APN311-202 trial (cohorts V1+V2 and V3 cohort) were available [35,38]. The DB studies enrolled patients from a broader population than the NAXI studies, including patients without evidence of disease at baseline or patients with disease outside bone and bone marrow. In the first step, patients with disease in soft tissues, those without evidence of disease at baseline (CR), and those treated during high-risk front-line therapy (APN311-202 trial, cohorts V1+V2 only) were excluded (NAXI is only registered in the treatment of relapsed or refractory disease in bone or bone marrow with PR, MR or SD). Furthermore, one patient with a local INSS stage 1, although he presented with disseminated metastasis prior to enrolment, was excluded from the V3 cohort of the APN311-202 trial (NAXI trials enrolled only patients with INSS stage 3 or higher; Figure 1). Both PFS and EFS data were available from the DB studies, but because only PFS was available for NAXI, EFS was not analysed.

Finally, data on 77 patients from DB studies were included in the comparison with the NAXI population.

### 3.3. MAIC of PFS

The comparison of the patient characteristics before and after adjustment for PFS (Study 201 vs. DB studies) is presented in Table 2. The PFS outcomes from Study 230 were not available for inclusion.

**Figure 2:**
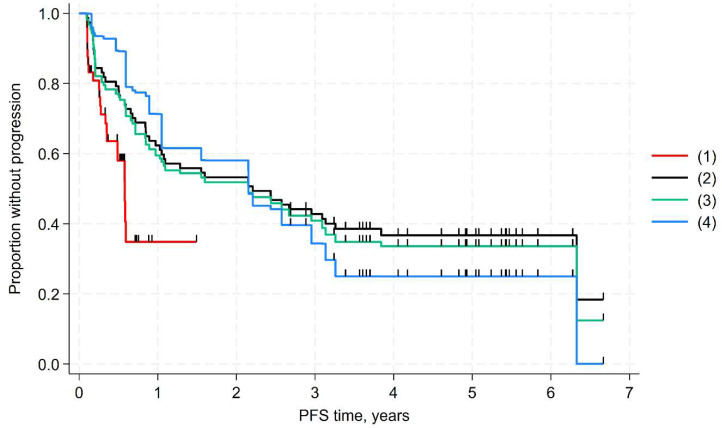
MAIC of PFS (DB with or without IL-2). Kaplan–Meier plots of compared arms: (1) naxitamab arm (Study 201); (2) DB unadjusted (Studies APN311-304, APN311-202, V1+V2 and APN311-202, V3); (3) DB arm weighted with variables: refractory, female, MYCN amplification, bone marrow only, bone and bone marrow (base-case analysis); (4) DB arm weighted with additional variables in MAIC: prior radiotherapy, Black race, % MYCN missing, % INSS stage 3 and % missing INSS (sensitivity analysis #1).

**Figure 3:**
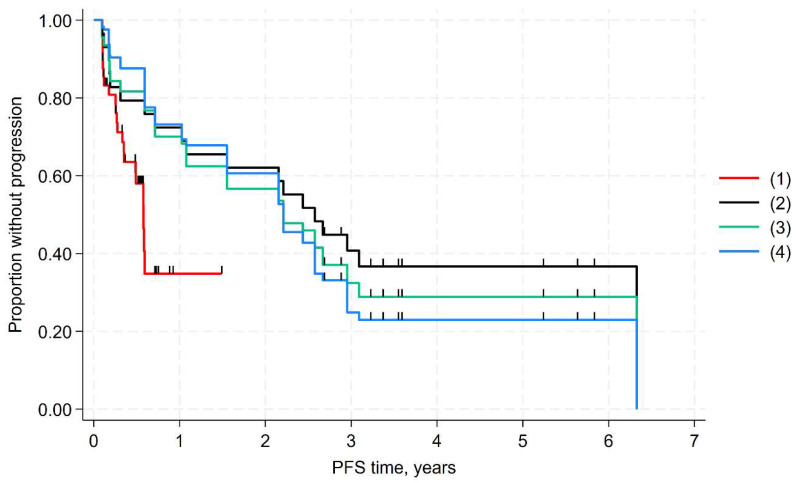
MAIC of PFS (DB without IL-2): (1) naxitamab arm (Study 201); (2) DB without IL-2 unadjusted (sensitivity analysis #2); (3) DB without IL-2 arm weighted with variables: refractory, female, MYCN amplification, bone marrow only, bone and bone marrow (sensitivity analysis #3); (4) DB without IL-2 arm weighted with additional variables in MAIC: prior radiotherapy, Black race, % MYCN missing, % INSS stage 3 and % missing INSS (sensitivity analysis #4).

The results of the base-case comparison revealed that DB ± IL-2 significantly extended PFS compared to patients treated with NAXI+GM-CSF (*p* = 0.015). The results of the unadjusted comparisons and the sensitivity analyses were consistent with the base case (Table 3, Figure 2 and Figure 3). The benefit of DB without IL-2 was similar to that of DB with or without IL-2.

### 3.4. MAIC of ORR

A comparison of patients’ characteristics before and after the adjustment is presented in Table 4.

The results of the base-case comparison revealed that DB ± IL-2 significantly improved ORR compared to NAXI + GM-CSF (*p* = 0.044). The results of unadjusted comparison were fully consistent with the results of the base case, but all sensitivity analyses (incorporating a lower number of patients than in the base case) indicate a non-significantly improved ORR in the DB arm compared to the NAXI arm. The benefit of DB without IL-2 was similar to that of DB with or without IL-2 (Table 5).

## 4. Discussion

Despite a growing number of clinical and real-world studies, there has been no direct comparison between the two anti-GD2 antibodies DB (±IL-2) and NAXI (+GM-CSF) in patients with relapsed/refractory neuroblastoma. Therefore, the aim of this systematic review was to identify data that allow the indirect comparison of the efficacy of the two treatments in a population of patients with relapsed/refractory neuroblastoma according to the approved narrower indication for NAXI—that is, patients with stable disease, a minor response or a partial response and disease in bone or bone marrow—based on the best available data.

Indirect treatment comparisons are based on the assumption of similarity between studies to produce unbiased estimates of the relative efficacy of treatments [40,41]. The lack of relevant RCTs resulted in the absence of a common comparator (anchor treatment); hence, indirect unanchored comparison was the only available method. However, IPD from the APN311-304 trial and from two phases of the APN311-202 trial (cohorts V1+V2 and cohort V3) were available for DB [35,38]. Therefore, harmonised inclusion criteria could be applied to DB populations to reflect the inclusion criteria of NAXI studies, making a reliable comparison possible.

Our study is the first comparison of NAXI and DB in the treatment of patients in the target population for NAXI to date. We included all the available data identified in the systematic review of medical databases. Additionally, we performed a two-stage adjustment of patients from DB trials: the selection of patients who met the inclusion criteria for the NAXI studies (with the exclusion of patients with CR after initial treatment or during front-line treatment, and those with disease in regions other than bone and bone marrow), and the adjustment of those patients for selected baseline characteristics (MYCN amplification, refractory disease, disease site and sex).

The results of our study indicate that DB ± IL-2 is more effective than NAXI+GM-CSF in the treatment of patients with relapsed or refractory neuroblastoma in the bone or bone marrow who have demonstrated a partial response, a minor response or stable disease following previous therapy. Base-case analysis and sensitivity analyses revealed that DB ± IL-2 significantly extended PFS compared to NAXI+GM-CSF. It should be noted that PFS is an outcome that is not affected by subsequent therapies and is assessed based on objective quantitative criteria. PFS is considered a sufficient outcome for assessing the efficacy of oncological drugs in the EMA [42] and FDA [43] registration process. While DB studies collected data for analysing both PFS and EFS, only PFS was available for NAXI, and as a result, only PFS could be used in the comparison. However, in relapsed/refractory NBL patients who have a high rate of progression and relatively low risk of secondary malignancies over the duration of clinical studies, particularly when high-dose chemotherapy is not used [44], the two outcomes can be considered very similar in future comparisons. Base-case analysis indicated that DB ± IL-2 also has a significantly higher ORR compared to NAXI+GM-CSF. Sensitivity analyses confirmed higher odds of having ORR in the DB arm than in the NAXI arm, but the difference did not reach the level of statistical significance, probably due to the lower sample sizes of most sensitivity analyses. Sensitivity analyses regarding the concomitant use of IL-2 indicate that the benefit of DB without IL-2 was similar to that of DB with IL-2.

Overall survival (OS), defined as the time from patient randomisation to death, is the gold standard for assessing the clinical benefit of cancer therapy [45]. This endpoint is easy to assess and is not prone to being affected by subjective interpretation by the investigator. However, it is affected by subsequent therapies [46]. In this case, information on therapies administered after relapse was not available from the identified sources. As a result, there were no data that could be used to balance the patient populations for treatments used after progression. Furthermore, demonstrating the clinical benefit in OS requires much larger sample sizes and a longer follow-up period compared to PFS [43]. A reliable comparison of OS between DB and NAXI was not feasible at the time of our study. Additionally, the OS data currently available for NAXI patients are immature (11.5% in Study 201 vs. more than 40% in the DB trials) [29,30,31,32,35,38]. Using available OS data, we can show that there was no statistically significant difference in OS between DB and NAXI in both unadjusted (*p* = 0.174) and adjusted comparisons (*p* = 0.096 to 0.615, Supplementary Table S16). However, these results do not reflect results from other endpoints (PFS and ORR) and are affected by low data maturity, loss to follow-up and different subsequent treatments in the compared arms.

Our study has other limitations that should be taken into account when interpreting the results: (i) Study 201 is in progress, with interim data only (https://clinicaltrials.gov/study/NCT03363373, accessed on: 15 April 2025), while other trials are complete. (ii) The duration of follow-up for patients in the DB arm vs. the NAXI arm differs. (iii) The reported data on patients from Study 201 are limited (no detailed information on baseline patient characteristics enabling the inclusion of more variables into the MAIC and/or performing more complex calculations).

Furthermore, there are differences in the baseline characteristics of the patients between the DB trial and the NAXI trials (for example, the proportions of races or ethnic groups, missing MYCN data, those with previous stem cell transplantation, missing INSS stage and INSS stage 3), which cannot be adjusted due to the low number of patients with these characteristics in the DB trials (1, 2 or 4 patients among the 77 included in the analyses). Adjustment for previous stem cell transplantation was not possible due to the insufficient number of patients treated with DB without prior SCT. As anti-GD2 antibody use without prior SCT is associated with poorer PFS (but not OS) [47], the difference in SCT use (27% in Study 201, 42% in Study 230 and 96% in DB studies) could have contributed to better outcomes in the DB arm. Additionally, adjustment for prior SCT should account for treatment line (first or after first). In the DB trials, 79.2% of all relapsed and refractory patients received HDT+SCT in the first line, while, among the relapsed patients, only 18.4% received HDT+SCT prior to DB but after relapse. In the NAXI Study 201, 30% of patients had prior SCT, but information by treatment line was not available. Furthermore, no patients in this matching cohort were treated with anti-GD2 immunotherapy prior to inclusion in DB studies (in comparison to 39% in NAXI studies), which could have contributed to the different outcomes, although there is no evidence demonstrating loss of efficacy on retreatment with anti-GD2 antibodies. It has been proposed that subsequent exposure might enhance anti-tumour activity [48,49], but supporting evidence is lacking.

The time from diagnosis to the first relapse is also an important predictor of survival outcomes [50,51]. However, it was not possible to adjust for this variable because only the median value was reported for NAXI (22 months), while the mean value would be more appropriate. There is a significant difference between the median and mean values for DB (35 and 48 months, respectively), so the two measures cannot be considered similar. Additionally, this variable is only relevant for relapsed patients, not refractory patients, and analysis could only be performed on the combined population because there was no subgroup data for relapsed patients for NAXI only. Consequently, excluding time from diagnosis to first relapse likely introduced a bias in the results, as patients in the DB studies had their first relapse approximately 13 months later than those in the NAXI study. Patients with earlier relapse have poorer prognosis. In contrast, earlier initiation of anti-GD2 immunotherapy is likely to lead to better outcomes [52,53,54]. The median time from last relapse to study entry was 6 months in Study 201 [30], while in the pooled DB data, it was 10 months. Local differences in time from actual progression to the detection of progression, which could have resulted from the frequency of scanning after first-line therapy, could also have affected outcomes in the compared groups.

Despite limitations, the presented MAIC results constitute the only currently available results for the comparison of DB and NAXI in the population of patients with relapsed or refractory neuroblastoma in bone or bone marrow who have demonstrated a partial response, a minor response or stable disease following previous therapy.

## 5. Conclusions

Results of the indirect comparison of dinutuximab beta and naxitamab were in favour of the former. Despite limitations, dinutuximab beta significantly increased overall response rate (ORR OR = 1.97, 95% CI: 1.02 to 3.80, *p* = 0.044) and significantly extended progression-free survival time (PFS HR = 0.47, 95% CI: 0.26 to 0.87, *p* = 0.015) compared to treatment with naxitamab.

## Figures and Tables

**Table 1:** Studies on relapsed or refractory neuroblastoma excluded from the MAIC.

Treatment	Study	Reasons
**Naxitamab**	Study 2PR01 (compassionate use) [32]	Retrospective Efficacy results presented for 6 patients only (ORR only; no PFS; no OS data) Safety data for 19 patients, including 13 from a different population Low expected number of patients from target population (<10) Compassionate use (treatment or patient selection unknown and/or not representative of whole population)
	Phase I of study 12-230 (NCT01757626) [32] **(phase II of this study was included in the MAIC)**	Dose-escalation design, resulting in only 6 patients treated with recommended dose of naxitamab, which is too small a number to allow for a meaningful comparison No baseline characteristic for cohort treated with approved dose of naxitamab Low expected number of patients from target population (<10)
**Dinutuximab beta**	Wieczorek et al., 2023 [33]	Retrospective Most patients had CR before DB treatment Low expected number of patients from target population (<10)
	APN311-201 (Flaadt et al., 2023) [36]	Low expected number of patients from target population (<10) Inappropriate dosing (28-day cycle) Inappropriate treatment (haplo-SCT required as treatment of all patients)
	Mueller et al., 2018 [37]/APN311-303 (compassionate use) [17]	Retrospective Low expected number of patients from target population (<10) Compassionate use (treatment or patient selection unknown and/or not representative of whole population)
	APN311-101 (Ladenstein et al., 2013) [39]	Retrospective Inappropriate dosing (28-day cycle; max 3 cycles; only 10 patients with recommended dose) Low expected number of patients from target population (<10)

DB—dinutuximab beta; CR—complete response; MAIC—matched-adjusted indirect comparison; OS—overall survival; ORR—overall response rate; PFS—progression-free survival; SCT—stem cell transplantation.

**Table 2:** MAIC of PFS (APN311-304 and APN311-202 vs. Study 201): patients’ characteristics.

Variable	Naxitamab, Study 201 (*n* = 52)	DB (*n* = 77) Before Weighting	*p* Value *	DB (*n* = 77) After Weighting	*p* Value *
Age, years	Median: 6 Range: 2–18	Median: 6.0 Mean: 6.61 Range: 2–19	-	Median: 6.0 Mean: 6.43 Range: 2–19	-
% refractory (n/N)	50.0% (26/52)	50.7% (39/77)	0.942	50.0%	>0.999
% female (n/N)	40.4% (21/52)	35.1% (27/77)	0.540	40.4%	>0.999
Prior treatment					
% prior stem cell transplant (n/N)	26.9% (14/52)	96.1% (74/77)	**<0.001**	95.3%	**<0.001**
% prior radiotherapy (n/N)	40.4% (13/52)	68.4% (52/77)	**<0.001**	70.7%	**<0.001**
% prior surgery (n/N)	88.5% (46/52)	90.8% (69/77)	0.837	90.9%	0.659
Disease site					
% bone only (n/N)	55.8% (29/52)	64.9% (50/77)	0.295	55.8%	>0.999
% bone marrow only (n/N)	3.8% (2/52)	9.1% (7/77)	0.251	3.8%	>0.999
% both (n/N)	40.4% (21/52)	26.0% (20/77)	0.085	40.4%	>0.999
Race/Ethnic origin					
% White (n/N)	34.6% (18/52)	85.7% (66/77)	**<0.001**	85.7%	**<0.001**
% Black (n/N)	3.8% (2/52)	2.6% (2/77)	0.688	2.1%	0.570
% Asian (n/N)	55.8% (29/52)	1.3% (1/77)	**<0.001**	2.3%	**<0.001**
MYCN					
% amplification (n/N)	13.5% (7/52)	9.1% (7/77)	0.434	13.5%	>0.999
% missing (n/N)	13.5% (7/52)	2.6% (2/77)	**0.018**	1.8%	**0.011**
INSS, diagnosis					
% stage 3 (n/N)	7.7% (4/52)	1.3% (1/77)	0.065	1.7%	0.109
% stage 4 (n/N)	88.5% (46/52)	97.4% (75/77)	**0.039**	97.4%	**0.047**
% missing (n/N)	3.8% (2/52)	1.3% (1/77)	0.346	0.9%	0.265

DB—dinutuximab beta; INSS—International Neuroblastoma Staging System; * Statistically significant differences between DB vs. NAXI are bolded.

**Table 3:** MAIC of PFS (APN311-304 and APN311-202 vs. Study 201): results of Cox model.

	Unadjusted Comparison	Base-Case MAIC ^A^	Sensitivity Analysis #1 ^B^	Sensitivity Analysis #2 ^C^	Sensitivity Analysis #3 ^D^	Sensitivity Analysis #4 ^E^
Log-rank test, *p* value *	**0.005**	-	-	**0.013**	-	-
HR (95% CI), *p* value *	0.44 (0.25 to 0.79), **0.006**	0.47 (0.26 to 0.87), **0.015**	0.28 (0.12 to 0.63), **0.002**	0.37 (0.16 to 0.83), **0.016**	0.36 (0.16 to 0.84), **0.017**	0.29 (0.12 to 0.69), **0.005**

CI—confidence interval; HR—hazard ratio; MAIC—matched-adjusted indirect comparison; PFS—progression free survival. * Statistically significant differences between DB vs. NAXI are bolded. ^A^ all patients from APN311-304 and APN311-202 trials (*n* = 77); MAIC with adjusted variables: refractory, female, MYCN amplification, bone marrow only, bone and bone marrow. ^B^ with additional variables in MAIC: prior radiotherapy, Black race, % MYCN missing, % stage 3 INSS and % missing INSS. ^C^ unadjusted comparison; patients without IL-2 treatment in DB arm (*n* = 29) vs. naxitamab in Study 201. ^D^ MAIC; patients without IL-2 treatment in DB arm (*n* = 29); adjusted variables: refractory, female, MYCN amplification, bone marrow only, bone and bone marrow. ^E^ MAIC; patients without IL-2 treatment in DB arm (*n* = 29); adjusted variables: refractory, female, MYCN amplification, bone marrow only, bone and bone marrow, prior radiotherapy, Black.

**Table 4:** MAIC of ORR: patients characteristics.

Variable	Naxitamab (*n* = 90), Study 201 and Study 230	DB (*n* = 77) Before Weighting	*p* Value *	DB (*n* = 77) After Weighting	*p* Value *
Age, years	Median: 6 (Study 201) Median: 5 (Study 230) Range: 2–23	Median 6.0 Mean 6.61 Range: 2–19	-	Median 6.0 Mean 6.48 Range: 2–19	-
% refractory (n/N)	47.8% (43/90)	50.7% (39/77)	0.711	47.8%	>0.999
% female (n/N)	44.4% (40/90)	35.1% (27/77)	0.218	44.4%	>0.999
Prior treatment					
% prior stem cell transplant (n/N)	33.3% (30/90)	96.1% (74/77)	**<0.001**	95.3%	**<0.001**
% prior radiotherapy (n/N)	34.4% (31/90)	68.4% (52/77)	**<0.001**	69.9%	**<0.001**
% prior surgery (n/N)	93.3% (84/90)	90.8% (69/77)	0.387	91.4%	0.643
Disease site					
% bone only (n/N)	53.3% (48/90)	64.9% (50/77)	0.129	53.3%	>0.999
% bone marrow only (n/N)	6.7% (6/90)	9.1% (7/77)	0.560	6.7%	>0.999
% both (n/N)	40.0% (36/90)	26.0% (20/77)	0.056	40.0%	>0.999
Race/Ethnic origin					
% White (n/N)	51.1% (46/90)	85.7% (66/77)	**<0.001**	86.4%	**<0.001**
% Black (n/N)	4.4% (4/90)	2.6% (2/77)	0.523	2.1%	0.410
% Asian (n/N)	35.6% (32/90)	1.3% (1/77)	**<0.001**	2.3%	**<0.001**
MYCN					
% amplification (n/N)	14.4% (13/90)	9.1% (7/77)	0.288	14.4%	>0.999
% missing (n/N)	12.2% (11/90)	2.6% (2/77)	**0.021**	1.5%	**0.011**
INSS, diagnosis					
% stage 3 (n/N)	5.6% (5/90)	1.3% (1/77)	0.141	1.7%	0.212
% stage 4 (n/N)	91.1% (82/90)	97.4% (75/77)	0.088	97.5%	0.092
% missing (n/N)	3.3% (3/90)	1.3% (1/77)	0.391	0.7%	0.270

DB—dinutuximab beta; INSS—International Neuroblastoma Staging System. * Statistically significant differences between DB vs. NAXI are bolded.

**Table 5:** MAIC of ORR: results.

	Unadjusted	Base-Case MAIC ^A^	Sensitivity Analysis #1 ^B^	Sensitivity Analysis #2 ^C^	Sensitivity Analysis #3 ^D^	Sensitivity Analysis #4 ^E^	Sensitivity Analysis #4 ^F^	Sensitivity Analysis #5 ^G^
ORR, naxitamab (95% CI)	43.3% (33.1% to 53.6%)	43.3% (33.1% to 53.6%)	43.3% (33.1% to 53.6%)	43.3% (33.1% to 53.6%)	43.3% (33.1% to 53.6%)	43.3% (33.1% to 53.6%)	50% (36% to 64%)	50.0% (36.4% to 63.6%)
ORR, dinutuximab beta (95% CI)	61.04% (47.10% to 74.98%)	60.1% (48.5% to 71.6%)	58.2% (41.8% to 74.5%)	58.62% (40.04% to 77.20%)	62.3% (43.7% to 80.9%)	64.6% (45.0% to 84.1%)	61.04% (47.10% to 74.98%)	60.4% (49.0% to 71.9%)
OR (95% CI)	2.05 (1.10 to 3.81)	1.97 (1.02 to 3.80)	1.82 (0.74 to 4.49)	1.85 (0.79 to 4.34)	2.16 (0.85 to 5.47)	2.38 (0.88 to 6.46)	1.57 (0.77 to 3.20)	1.53 (0.73 to 3.20)
*p* value *	**0.024**	**0.044**	0.195	0.156	0.105	0.088	0.218	0.263

CI—confidence interval; MAIC—matched-adjusted indirect comparison; OR—odds ratio; ORR—overall response rate. * Statistically significant differences between DB vs. NAXI are bolded. ^A^ all patients from DB studies (APN311-304 and APN311-202); NAXI patients from Study 201 and Study 230; MAIC with adjusted variable: refractory, female, MYCN amplification, bone marrow only, bone and bone marrow. ^B^ with additional variables in MAIC: prior radiotherapy, Black, MYCN missing, INSS = 3, INSS missing. ^C^ unadjusted comparison; patients without IL-2 treatment in DB arm (*n* = 29) vs. NAXI in Study 201 and Study 230. ^D^ patients without IL-2 treatment from DB studies; NAXI patients from Study 201 and Study 230; MAIC with adjusted variable: refractory, female, MYCN amplification, bone marrow only, bone and bone marrow. ^E^ SA#3 with additional MAIC variables: prior radiotherapy, Black race. ^F^ unadjusted comparison; only Study 201. ^G^ all patients from DB studies; NAXI patients from Study 201; MAIC with adjusted variable: refractory, female, MYCN amplification, bone marrow only, bone and bone marrow.

## Supplementary Materials

The following supporting information can be downloaded at: https://www.mdpi.com/article/10.3390/cancers17172723/s1, Figure S1: Search flow diagram; Table S1: PubMed—last search for dinutuximab beta: 15.04.2025; Table S2: Cochrane Library —last search for dinutuximab beta: 15.04.2025 (all text); Table S3: EMBASE—last search for dinutuximab beta: 15.04.2025, Table S4: Other sources—last search for dinutuximab beta: 15.04.2025; Table S5: PubMed—last search for naxitamab: 15.04.2025; Table S6: Cochrane Library—last search for naxitamab: 15.04.2025 (all text); Table S7: EMBASE—last search for naxitamab: 15.04.2025; Table S8: Other sources—last search for naxitamab: 15.04.2025; Table S9: Inclusion and exclusion criteria for systematic review; Table S10: Registered indication and dosing for dinutuximab beta and naxitamab in neuroblastoma treatment; Table S11: Methodology of identified studies for naxitamab in relapse/refractory neuroblastoma; Table S12: Methodology of identified studies for dinutuximab beta in relapse/refractory neuroblastoma; Table S13: Assessment of prospective single-arm studies according to NICE criteria—part I; Table S14: Assessment of prospective single-arm studies according to NICE criteria—part II; Table S15: Risk of Bias 2.0 assessment APN311-202 Phase II—RCT; Table S16: MAIC of OS: results.
