# Supplementary material for: Dinutuximab Beta Versus Naxitamab in the Treatment of Relapsed/Refractory Neuroblastoma in Patients with Stable Disease, Minor Response or Partial Response and Disease in Bone or Bone Marrow: Systematic Review and Matching-Adjusted Indirect Comparison
Source: Cancers (Basel). 2025 Aug 22;17(17):2723. doi: 10.3390/cancers17172723 (PMC12427561; doi:10.3390/cancers17172723)
Supplement: Supplementary file 1 [file cancers-17-02723-s001.zip › cancers-3808240-supplementary.pdf]

Supplement

Supplementary Table S1. PubMed – last search for dinutuximab beta: 15.04.2025.

Number	Keywords	Search results
**#1**	neuroblastoma	51 196
**#2**	relapse* OR refractory OR salvage OR "bridge therapy" OR recurrent OR resistant	2 721 567
**#3**	dinutuximab OR ch14.18 OR ch14.18/CHO OR Qarziba OR Dinutuximab beta	330
**#4**	#1 AND #2 AND #3	**110**

Supplementary Table S2. Cochrane Library - last search for dinutuximab beta: 15.04.2025 (all text).

Number	Keywords	Search results
**#1**	neuroblastoma	666
**#2**	relapse* OR refractory OR salvage OR "bridge therapy" OR recurrent OR resistant	239 200
**#3**	dinutuximab OR ch14.18 OR ch14.18/CHO OR Qarziba OR Dinutuximab beta	77
**#4**	#1 AND #2 AND #3	38
**#5**	#4 *	**37**

*filters: register of clinical trials

Supplementary Table S3. EMBASE – last search for dinutuximab beta: 15.04.2025.

Number	Keywords	Search results
**#1**	neuroblastoma	72 095
**#2**	relapse* OR refractory OR salvage OR "bridge therapy" OR resistant	1 530 704
**#3**	dinutuximab AND beta OR dinutuximab OR ch14.18 OR (ch14.18 AND cho) OR qarziba	1 031
**#4**	#1 AND #2 AND #3	312
**#5**	#4*	**195**

* AND [embase]/lim NOT [medline]/lim

Supplementary Table S4. Other sources – last search for dinutuximab beta: 15.04.2025.

Website	Keywords	Search results
**EMA (https://www.ema.europa.eu/en/homepage)**	Dinutuximab beta	8
**FDA (https://www.fda.gov/)**	Dinutuximab beta	15

Supplementary Table S5. PubMed – last search for naxitamab: 15.04.2025.

Number	Keywords	Search results
**#1**	neuroblastoma	51 196
**#2**	relapse* OR refractory OR salvage OR "bridge therapy" OR recurrent OR resistant	2 721 567
**#3**	Naxitamab OR danyelza OR hu3F8	66
**#4**	#1 AND #2 AND #3	**33**

Supplementary Table S6. Cochrane Library - last search for naxitamab: 15.04.2025 (all text).

Number	Keywords	Search results
**#1**	neuroblastoma	664
**#2**	relapse* OR refractory OR salvage OR "bridge therapy" OR recurrent OR resistant	238 714
**#3**	Naxitamab OR danyelza OR hu3F8	10
**#4**	#1 AND #2 AND #3	7
**#5**	#4 *	**6**

*filters: register of clinical trials

Supplementary Table S7. EMBASE – last search for naxitamab: 15.04.2025.

Number	Keywords	Search results
#1	neuroblastoma	70 779
#2	relapse* OR refractory OR salvage OR "bridge therapy" OR resistant	1 486 168
#3	Naxitamab OR danyelza OR hu3F8	200
#4	#1 AND #2 AND #3	83
#5	#4*	**59**

* AND [embase]/lim NOT [medline]/lim

Supplementary Table S8. Other sources – last search for naxitamab: 15.04.2025.

Website	Keywords	Search results
**EMA (https://www.ema.europa.eu/en/homepage)**	Naxitamab	1
**FDA (https://www.fda.gov/)**	Naxitamab	23
**ISPOR (https://www.ispor.org/heor-resources/presentations-database/presentation/euro2024-4015/141758)**	Naxitamab	3
**ESMO (https://www.esmo.org/)**	Naxitamab	5
**ASCO (https://ascopubs.org/)**	Naxitamab	3
**LARVOL (https://clin.larvol.com)**	Naxitamab	23

Supplementary Table S9. Inclusion and exclusion criteria for systematic review.

	Inclusion criteria	Exclusion criteria
**Population**	Relapsed and/or refractory and/or recurrent neuroblastoma in patients aged 12 months and above	Patients with other tumours, newly diagnosed neuroblastoma treated in maintenance
**Intervention and comparators**	Intervention: Dinutuximab beta used in maintenance therapy, in combination with IL-2 or as single agent (as recommended by SIOPEN), dosing according to approved dosing<br><br>Comparator: naxitamab + GM-CSF*; dosing according to FDA prescribing information.	Other options, dinutuximab beta or naxitamab used with chemotherapy combinations (chemoimmunotherapy) were excluded
**Outcomes**	EFS – event free survival<br>PFS – progression free survival<br>OS – overall survival<br>ORR – overall response rate.	Trials not reported defined outcomes
**Study types**	Randomized controlled trials, non-randomized studies with control group, large observational, single-arm studies (with ≥10 patients)	Reviews, additional analysis of included trials, small observational studies, case reports
**Publication type**	Clinical study reports, full text articles or data from registration documents (EPAR, SmPC), in the absence of any references published as full texts, inclusion of abstracts (posters and conference presentations) with most recent results was allowed	Editorials, letters, data from clinical trials registers, reviews
**Language**	No restrictions	-

*For naxitamab: trials on patients with complete response or progressive disease before use of this drug were excluded (according to the approved indication).

Supplementary Table S10. Registered indication and dosing for dinutuximab beta and naxitamab in neuroblastoma treatment.

	Dinutuximab beta (Qarziba®) https://www.ema.europa.eu/en/documents/product-information/qarziba-epar-product-information_en.pdf	Naxitamab (Danyelza®) https://www.accessdata.fda.gov/drugsatfda_docs/label/2020/761171lbl.pdf
**Registered indication**	By EMA:	By FDA:

	**Dinutuximab beta (Qarziba®) https://www.ema.europa.eu/en/documents/product-information/qarziba-epar-product-information_en.pdf**	**Naxitamab (Danyelza®) https://www.accessdata.fda.gov/drugsatfda_docs/label/2020/761171lbl.pdf**
	Qarziba® is indicated for the treatment of high-risk neuroblastoma in patients aged 12 months and above, who have previously received induction chemotherapy and achieved at least a partial response, followed by myeloablative therapy and stem cell transplantation, **as well as patients with history of relapsed or refractory neuroblastoma, with or without residual disease. Prior to the treatment of relapsed neuroblastoma, any actively progressing disease should be stabilised by other suitable measures.** **In patients with a history of relapsed/refractory disease and in patients who have not achieved a complete response after first line therapy, Qarziba® should be combined with interleukin-2 (IL-2)^**	DANYELZA® is indicated, in combination with granulocyte-macrophage colony-stimulating factor (GM-CSF), for the treatment of pediatric patients 1 year of age and older and adult patients with relapsed or refractory high-risk neuroblastoma in the bone or bone marrow who have demonstrated a partial response, minor response, or stable disease to prior therapy*.
**Dosing**	Treatment with Qarziba® consists of 5 consecutive courses, each course comprising 35 days. The individual dose is determined based on the body surface area and should be a total of 100 mg/m^2 per course.<br><br>Two modes of administration are possible:<br>• a continuous infusion over the first 10 days of each course (a total of 240 hours) at the daily dose of 10 mg/m^2;<br>• or five daily infusions of 20 mg/m^2 administered over 8 hours, on the first 5 days of each course.<br><br>When IL-2 is combined with Qarziba®, it should be administered as subcutaneous injections of 6×10 6 IU/m^2 /day, for 2 periods of 5 consecutive days, resulting in an overall dose of 60×106 IU/m^2 per course. The first 5-day course should start 7 days prior to the first infusion of dinutuximab beta and the second 5-day course should start concurrently with dinutuximab beta infusion (days 1 to 5 of each dinutuximab beta course).	The recommended dosage of DANYELZA® is 3 mg/kg/day (up to 150 mg/day) on Days 1, 3, and 5 of each treatment cycle, administered as an intravenous infusion after dilution.<br><br>Treatment cycles are repeated every 4 weeks until complete response or partial response, followed by 5 additional cycles every 4 weeks. Subsequent cycles may be repeated every 8 weeks. Discontinue DANYELZA and GM-CSF for disease progression or unacceptable toxicity. Administer pre-infusion medications and supportive treatment, as appropriate, during infusion.

EMA – European Medicines Agency; FDA – Food and Drug Administration; GM-CSF – granulocyte-macrophage colony stimulating factor; IL-2 – interleukin-2.
^ IL-2 is not recommended by treatment guidelines.
* naxitamab is not recommended for treatment of patients with soft tissue disease based on guidelines/recommendations.

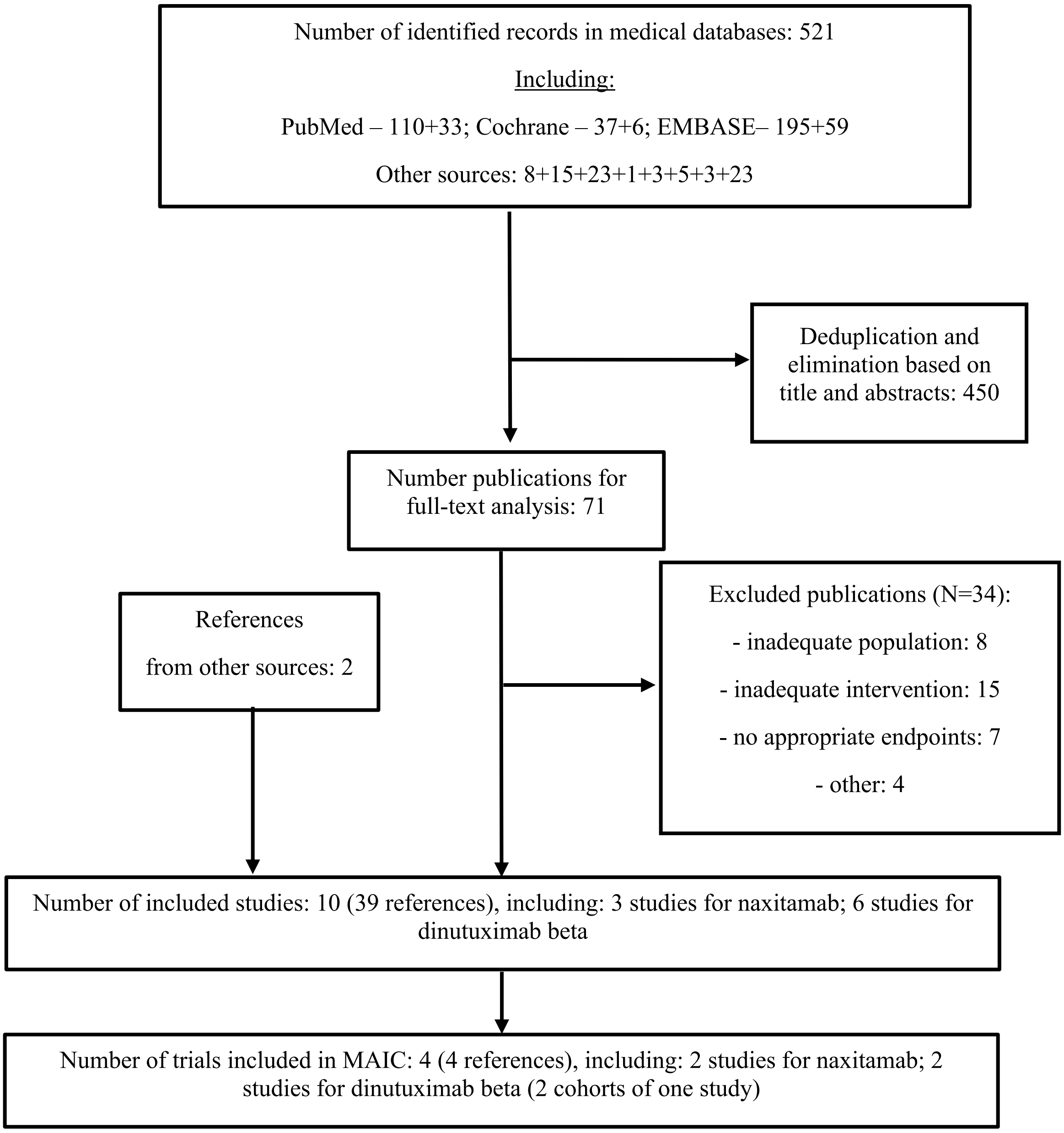

Supplementary Figure S1. Search flow diagram.

Bibliography of references of all included studies in broad systematic review (all trials included in review and MAIC) - key publications for each study are marked in bold:

__Included studies for dinutuximab beta__

[1] Wieczorek A, Żebrowska U, Ussowicz M et al. Dinutuximab Beta Maintenance Therapy in Patients with High-Risk Neuroblastoma in First-Line and Refractory/Relapsed Settings—Real-World Data. J. Clin. Med. 2023, 12, 5252. https://doi.org/10.3390/jcm12165252.

[2] Lode H, Ehlert K, Huber S et al. Long-term, continuous infusion of single-agent dinutuximab beta for relapsed/refractory neuroblastoma: an open-label, single-arm, Phase 2 study. British Journal of Cancer; 2023;12(19):6196. https://doi.org/10.1038/s41416-023-02457-x

[3] CSR and individual patient data for study 304

[4] Flaadt T, Landstein RL, Ebinger M et al. Anti-GD2 Antibody Dinutuximab Beta and Low-Dose Interleukin 2 After Haploidentical Stem-Cell Transplantation in Patients With Relapsed Neuroblastoma: A Multicenter, Phase I/II Trial. J Clin Oncol. 2023 Jun 10;41(17):3135-3148.

[5] CSR and individual patient data from study 202

[6] Mueller I, Ehlert K, Endres S et al. Tolerability, response and outcome of high-risk neuroblastoma patients treated with long-term infusion of anti-GD2 antibody ch14.18/CHO. MABS 2018, 10(1), 55–61.

[7] Ladenstein R, Weixler S, Baykan B et al. Ch14.18 antibody produced in CHO cells in relapsed or refractory Stage 4 neuroblastoma patients: a SIOPEN Phase 1 study. MAbs. 2013 Sep-Oct;5(5):801-9.

[8] EMA EPAR 2017 https://www.ema.europa.eu/en/documents/assessment-report/dinutuximab-beta-apeiron-epar-public-assessment-report_en.pdf; accessed: July 2024

__Included studies for naxitamab__

[9] CENTER FOR DRUG EVALUATION AND RESEARCH. Application number 761171Orig1s000. DANYELZA, https://www.accessdata.fda.gov/drugsatfda_docs/nda/2020/761171Orig1s000TOC.cfm naxitamab-gqgk

[10] Morgenstern, D.A., Mora, J., Chan, G.C et al. 74P Pivotal trial 201 data on outpatient administration of naxitamab (Hu3F8), a humanized GD2 targeted immunotherapy for the treatment of refractory/relapsed (R/R) high-risk (HR) neuroblastoma (NB). Ann. Oncol. 2020 - Volume 31, Issue 0, pp. S1448. https://www.annalsofoncology.org/article/S0923-7534(20)43075-3/fulltext

[11] Mora, J., Chan, G.C., Morgenstern, D.A et al. 75P Efficacy and updated safety results from pivotal phase II trial 201 of naxitamab (Hu3F8): A humanized GD2-targeted immunotherapy for the treatment of refractory/relapsed (R/R) high-risk (HR) neuroblastoma (NB). Ann. Oncol. - Volume 31, Issue 0, pp. S1448. https://www.annalsofoncology.org/article/S0923-7534(20)43076-5/fulltext

[12] Kushner, B.H., Morgenstern, D.A., Nysom et al. Efficacy of naxitamab in patients with refractory/relapse (R/R) highrisk neuroblastoma (HR-NB) by bone/bone marrow (BM) evaluation, potential sites of residual disease. J. Clin. Oncol. - Volume 39, Issue 15, pp. - published 2021. https://ascopubs.org/doi/10.1200/JCO.2021.39.15_suppl.10022

[13] Mora, J., Chan, G.C., Morgenstern, D.A et al. Naxitamab-related adverse events within and across treatment cycles in patients with relapsed/refractory (R/R) high-risk neuroblastoma. J. Clin. Oncol. 2024 Volume 42, Issue 16. https://ascopubs.org/doi/10.1200/JCO.2024.42.16_suppl.10032

[14] Mora J, Chan GCF, Morgenstern DA, Amoroso L, Nysom K, Faber J, Wingerter A, Bear MK, Rubio-San-Simon A, de Las Heras BM, Tornøe K, Düring M, Kushner BH. The anti-GD2 monoclonal antibody naxitamab plus GM-CSF for relapsed or refractory high-risk neuroblastoma: a phase 2 clinical trial. Nat Commun. 2025 Feb 14;16(1):1636. doi: 10.1038/s41467-025-56619-x. PMID: 39952926; PMCID: PMC11828896.

[15] Mora, J., Chan, G.C., Morgenstern, D.A et al. Patterns of improvement following initial response in patients treated with naxitamab for relapsed/refractory high-risk neuroblastoma. J. Clin. Oncol. - Volume 42, Issue 16, pp. - published 2024. https://ascopubs.org/doi/10.1200/JCO.2024.42.16_suppl.10033

[16] Kushner, B., Mora, J., Chan, G. et al. Naxitamab efficacy in patients with refractory/relapsed high-risk neuroblastoma and bone metastases as assessed by Curie score. Immuno-Oncology Technol. - Volume 20, Issue 0, pp. - published 2023 https://www.esmoiotech.org/article/S2590-0188(23)00264-2/fulltext

[17] Kushner, B., Chan, G., Morgenstern, D et al. REDUCTION IN BONE METASTASES AND CURIE SCORE IN PATIENTS TREATED WITH naxitamab IN TRIAL 201. Pediatr. Blood Cancer - Volume 70, Issue 0, pp. - published 2023.

[18] Marachelian, A., Watt, T., August, K et al. REAL-WORLD DATA FROM ADVERSE EVENTS ASSOCIATED WITH naxitamab TREATMENT IN THE UNITED STATES (US). Pediatr. Blood Cancer - Volume 70, Issue 0, pp. - published 2023.

[19] Mora, J., Chan, G.C., Morgenstern, D.A et al. 62MO Naxitamab pivotal clinical trial planned interim analysis of PFS and OS in patients with relapsed or refractory high-risk neuroblastoma. Immuno-Oncology Technol. - Volume 16, Issue 0, pp. - published 2022. https://www.esmoiotech.org/article/S2590-0188(22)00098-3/fulltext

[20] Mora, J., Bear, M., Chan, G et al. Naxitamab treatment for relapsed or refractory high-risk neuroblastoma: Outcomes from the first prespecified analyses of the Pivotal 201 Trial. Ann. Oncol. 2022 - Volume 33, Issue 0, pp. S956. https://www.annalsofoncology.org/article/S0923-7534(22)02868-X/fulltext

[21] Mora, J., Chan, G.C.-F., Morgenstern, D.A et al. Naxitamab (NAX) treatment for refractory/relapsed (R/R) high-risk neuroblastoma (HRNB): Response data and efficacy in patient (pt) subgroups. J. Clin. Oncol. - Volume 40, Issue 16, pp. - published 2022. https://ascopubs.org/doi/10.1200/JCO.2022.40.16_suppl.e22019

[22] Mora, J., Bear, M., Chan, G et al. Assessment of corticosteroid premedication for patients treated with the humanized GD2-binding monoclonal antibody naxitamab. Ann. Oncol. - Volume 32, Issue 0, pp. S1451.

[23] Mora, J., Bear, M., Chi-Fung Chan, G et al. Naxitamab Treatment of Refractory/Relapsed High-Risk Neuroblastoma (R/R HR NB); Subgroup Analysis of Updated Interim Efficacy and Safety Data for the Registrational Phase II Trial. Pediatr. Blood Cancer - Volume 68, Issue 0, pp. - published 2021. https://cslide.ctimeetingtech.com/siop21/attendee/confcal/show/session/123

[24] Mora J., Chan G.C., Morgenstern D.A., Amoroso L., Nysom K., Faber J., Wingerter A., Bear M., Rubio-San-Simon A., Tornøe K., Koep S., Düring M., Kushner B.H. Disease control in patients treated with naxitamab for refractory/relapsed high-risk neuroblastoma. Cancer Research 2024 84:17 Supplement

[25] Mora J., Chan G.C., Morgenstern D.A., Amoroso L., Nysom K., Faber J., Wingerter A., Bear M., Rubio-San-Simón A., Eldridge J., Tornøe K., Döuring M., Kushner B.H. Naxitamab-related adverse events within and across treatment cycles in patients with relapsed/refractory (R/R) high-risk neuroblastoma. Journal of Clinical Oncology 2024 42:16 Supplement

[26] Mora, J., Bear, M., Chan, G., Morgenstern, D.A et al. 963MO Naxitamab for the treatment of refractory/relapsed high-risk neuroblastoma (HR NB): Updated efficacy and safety data from the international, multicenter phase II trial 201. Ann. Oncol. 2021 - Volume 32, Issue 0, pp. S833. https://www.annalsofoncology.org/article/S0923-7534(21)03577-8/fulltext

[27] Mora, J., Chi-Fung Chan, G., Morgenstern, D.A et al. Naxitamab, a new generation anti-GD2 monoclonal antibody (mAb) for treatment of relapsed/refractory high-risk neuroblastoma (HR-NB). J. Clin. Oncol. - Volume 38, Issue 15, pp. - published 2020 https://ascopubs.org/doi/10.1200/JCO.2020.38.15_suppl.10543

[28] Mora, J., Castañeda, A., Colombo et al. Clinical and pathological evidence of anti-gd2 immunotherapy induced differentiation in relapsed/refractory high-risk neuroblastoma. Cancers (Basel). 2021 Mar 12;13(6):1264.

[29] Mora, J., Chan, G., Morgenstern, D., Nysom et al. International, multicenter phase ii trial with humanized anti-GD2 monoclonal antibody naxitamab for treatment of refractory/relapsed high-risk neuroblastoma: Efficacy and safety data. Pediatr. Blood Cancer - Volume 67, Issue 0, pp. - published 2020. https://ir.ymabs.com/static-files/d65ebb1e-efcf-453d-bbe1-69e44685f5db

[30] Mora J, Chan GC, Morgenstern DA et al. Outpatient administration of naxitamab in combination with granulocyte-macrophage colony-stimulating factor in patients with refractory and/or relapsed high-risk neuroblastoma: Management of adverse events . Cancer Rep (Hoboken). 2023 Jan;6(1):e1627.

[31] Castañeda A, Gorostegui M, Miralles SL et al. How we approach the treatment of patients with high-risk neuroblastoma with naxitamab: experience from the Hospital Sant Joan de Déu in Barcelona, Spain. ESMO Open 2022 Apr;7(2):100462.

[32] Mora et al. Conference presentation: A pivotal phase 2 trial of antibody naxitamab (hu3F8) and granulocyte-macrophage colony-stimulating factor (GM-CSF)inhigh-risk neuroblastoma patients with primary refractory disease or incomplete response to salvage treatment in bone and/or bone marrow(NCT03363373). ESMO 2021

[33] Kushner BH, Modak S, Basu EM et al. Safety and efficacy of naxitamab plus modified dosing of GM-CSF for patients with high-risk neuroblastoma (HR-NB) in first complete remission (CR) or with primary refractory disease. Journal of Clinical Oncology Volume 41, Number 16_suppl, 2023 https://ascopubs.org/doi/10.1200/JCO.2023.41.16_suppl.e22012

[34] Kushner B,· Chan GC,·Morgenstern DA et al. 109P Impact of anti-drug antibody (ADA) on naxitamab efficacy and safety. Immuno-Oncology and Technology Volume 16, Supplement 1, 100213, December 2022 https://www.esmoiotech.org/article/S2590-0188(22)00144-7/fulltext

[35] Larvol clin – results for naxitamab on the website results for naxitamab on the website https://clin.larvol.com/trial-detail/NCT03363373

[36] Kushner, B., Modak, S., Basu et al. High-dose naxitamab (humanized-3f8) plus stepped-up dosing of granulocyte-macrophage colony-stimulating factor (GM-CSF) for resistant osteomedullary neuroblastoma: Major responses and outpatient treatment in a phase ii trial. Pediatr. Blood Cancer - Volume 67, Issue 0, pp. - published 2020 https://ir.ymabs.com/static-files/5dcbd115-6eac-4316-8f20-67ab73df1f97

[37] Kushner, B.H., Modak, S., Basu, E.M et al. High-dose naxitamab plus stepped-up dosing of GM-CSF for high-risk neuroblastoma (HR-NB): Efficacy against histologically-evident primary refractory metastases in bone marrow (BM). J. Clin. Oncol. 2019 - Volume 37, https://ascopubs.org/doi/10.1200/JCO.2019.37.15_suppl.10024

[38] Kushner BH, Cheung IY, Modak S et al. Humanized 3F8 Anti-GD2 Monoclonal Antibody Dosing With Granulocyte-Macrophage Colony-Stimulating Factor in Patients With Resistant Neuroblastoma: A Phase 1 Clinical Study . JAMA Oncol. 2018 Dec 1;4(12):1729-1735.

[39] Cheung IY, Kushner BH, Modak S et al. Phase I trial of anti-GD2 monoclonal antibody hu3F8 plus GM-CSF: Impact of body weight, immunogenicity and anti-GD2 response on pharmacokinetics and survival. Oncoimmunology. 2017 Jul 31;6(11):e1358331.

Supplementary Table S11. Methodology of identified studies for naxitamab in relapse/refractory neuroblastoma.

Reference	Type of trial	Key inclusion criteria	Key exclusion criteria	Interventions (for patients with R/R disease)	Number of included patients
**Study 12-201 (NCT03363373) [9]-[35]**	Multicenter, single-arm, nonrandomized, open-label, Phase II trial	The trial enrolled subjects with neuroblastoma diagnosed by 1) histopathology of tumour biopsy or 2) bone marrow aspirate or biopsy indicative of neuroblastoma by histology plus high urine catecholamine levels or MYCN amplification or MIBG-avid lesion(s).<br><br>Enrolment was open to subjects older than 1 year of age with a diagnosis of neuroblastoma, as defined in the section on diagnostic criteria above. Subjects should have high-risk neuroblastoma with either primary refractory disease or incomplete response to salvage treatment, evaluable in bone and/or bone marrow; if disease was only present in bone, the subject should have evaluable disease outside the radiation areas for being eligible.<br>High-risk neuroblastoma (NB) was defined as INRG MYCN-amplified any INRG stage of any age, or MYCN-nonamplified with INRG Stage M subjects, diagnosed at 18 months of age or older.<br><br>Primary refractory disease was defined as no relapse or PD, but incomplete metastatic response (SD, MR, PR defined by INRC) to	Subjects were excluded for any systemic anticancer therapy, including chemotherapy or immunotherapy within 3 weeks before the first dose of GM-CSF. Subjects were also excluded for evaluable NBL outside bone and bone marrow sites.	**NAX+GM-CSF**<br><br>Naxitamab: dose 9.0 mg/kg per cycle administered on Days 1, 3, and 5 of each cycle (3.0 mg/kg per infusion) in the presence of s.c. GM-CSF (Days –4 through 5).<br><br>GM-CSF over a 10-day period in a cycle. A cycle started with 5 days of GM-CSF administered s.c. at 250 μg/m2/d before the start of naxitamab infusions (Day –4 to Day 0) (Figure 8-1). On Days 1 through 5, GM-CSF was administered s.c. at a dose of 500 μg/m2/d.<br>On Days 1, 3, and 5, naxitamab was infused i.v. over approximately 30 minutes.	The most recent data:<br>N=52 for efficacy analysis<br><br>N=74 for safety analysis

Reference	Type of trial	Key inclusion criteria	Key exclusion criteria	Interventions (for patients with R/R disease)	Number of included patients
		treatment in bone and/or BM as documented either by histologic evidence of NB in bone or BM and/or abnormal 123I-MIBG uptake in skeletal sites.<br>In addition, at trial enrollment, subjects could not be more than 18 months from initiation of chemotherapy and must have received at least 4 cycles of standard induction chemotherapy for high-risk NB. Incomplete response to salvage treatment was defined as relapse or PD, and incomplete response (SD, MR, PR, but not PD, defined by INRC) to salvage chemotherapy or MIBG therapy immediately before enrollment. Furthermore, it was restricted to lesions in bone and/or BM as documented either by histologic evidence of NB in bone or BM and/or abnormal 123I-MIBG uptake in skeletal sites. In addition, a minimum of 2 months from last MIBG/PET-CT documented relapse or PD prior to enrollment was required.<br><br>Two cohorts of subjects are enrolled in the trial:<br>• Cohort 1 includes subjects who are screened negative for ADA and with no preplanned radiotherapy for metastatic lesions.<br>• Cohort 2 includes subjects who are screened positive for ADA or with preplanned radiotherapy for metastatic lesions.			
**Study 12-230 (NCT01757626) [9], [36]-[39]**	Study 12-230 had two phases. Phase I was a single-center, single-arm, open-label, 3 + 3 dose-escalation design. Phase II was a single-center, single-arm, nonrandomized, open-label trial.	Subjects older than 1 year of age with a diagnosis of neuroblastoma, as defined in the section on diagnostic criteria (1) histopathology or 2) bone marrow metastases or MIBG-avid lesion(s) plus high urine catecholamine levels). Subjects must have had high-risk neuroblastoma (including MYCN-amplified stage 2/3/4/4s of any age and MYCN-nonamplified Stage 4 in subjects greater than 18 months or age).<br><br>Phase I:<br>subjects must have had refractory or relapsed neuroblastoma that was resistant to standard therapy (standard therapy included intensive induction chemotherapy, followed by a variety of consolidation or salvage therapies, depending on response).<br>Clinical status at enrollment had to be any of the following: primary refractory disease, defined as incomplete response to high-risk neuroblastoma therapy but no prior relapse or progressive disease; secondary refractory disease, defined as incomplete response to salvage therapy for prior relapse or progressive disease; current progressive disease; or second or later complete remission, ie, no assessable neuroblastoma after salvage therapy for prior relapse or progressive disease.<br><br>Phase II:<br>primary or secondary refractory disease in bone or bone marrow, defined as morphologic evidence of neuroblastoma in bone marrow and/or abnormal ^{123}I-MIBG uptake in osteomedullary sites, or had to	No chemotherapy or immunotherapy for a minimum of 3 weeks prior to start of naxitamab	**NAX+GM-CSF**<br><br>Phase I:<br>escalating doses of intravenous (i.v.) naxitamab infusions (Days 1, 3, 5) in combination with subcutaneous (s.c.) GM-CSF (Day –4 through 5) during a 10-day cycle repeated every 2 to 4 weeks (There were 15 dosage levels in this phase I trial, ranging from 0.9 mg/kg up to 9.6 mg/kg of total dose given per cycle). Subjects with no dose-limiting toxicity (as defined in protocol) and no progression of disease during the first cycle had the option of receiving an additional 3 cycles if antidrug antibody (ADA) titer was negative.<br>Subjects who completed 4 cycles of treatment without complications or disease progression had the option of continuing treatment for up to 24 months from their first dose of naxitamab.<br>The 3 + 3 dose-escalation design for phase 1 trials was used. Thus, 3 to 6 patients were treated at each dose level,	Phase I:<br>N=57 [38], [39] (≤6 patients with recommended dosing)<br><br>Phase II:<br>N=38

Reference	Type of trial	Key inclusion criteria	Key exclusion criteria	Interventions (for patients with R/R disease)	Number of included patients
		be in second or greater complete response as defined by INRC with 123I-MIBG findings quantitated by Curie scores. Prior treatment with other anti-GD2 antibody therapies, including murine 3F8, naxitamab, ch14.18, and hu14.18 was allowed.<br><br>In Phase 2, subjects were enrolled into one of three subject groups described as follows:<br>• Group 1: Subjects with primary refractory disease (no prior relapse, but incomplete response to treatment) in bone and/or bone marrow, as documented by histology and or 123I-MIBG scan.<br>• Group 2: Subjects in second CR or very good partial response and risk for another relapse. The VGPR criterium was removed from Group 2 enrollment criteria with Amendment 20 (**results only for safety analysis**).<br>• Group 3: Subjects with secondary refractory disease (prior relapse and incomplete response to retrieval therapy) in bone and/or bone marrow as documented by histology and/or 123I-MIBG. (Note: hereafter this will be referred to as incomplete response to salvage treatment.)<br><br>**Subjects from Group 2 had no tumour at baseline, therefore, this subject population is not part of the efficacy population but only contributes to the safety population.**<br>The two efficacy analysis groups are the FAS (full analysis set) and PAS (primary analysis set). Both groups enrolled subjects with baseline disease defined in Groups 1 and 3 (primary refractory disease and incomplete response to salvage therapy, had evaluable disease at baseline, and began at least one infusion of naxitamab)		and the maximum-tolerated dose was the highest level at which 0 of 3 or 1 of 6 patients experienced DLT<br><br>Phase II:<br>naxitamab at 9.0 mg/kg per cycle. Dosing with naxitamab occurred on Days 1, 3, and 5 of each cycle in the presence of s.c. GM-CSF (Days –4 through 5). Cycles were repeated monthly through 5 cycles, then every 1 to 2 months for up to 24 months from trial enrollment or until a subject had received five cycles after a CR or PR was achieved, whichever came first.	
**Study 2PR01 – compassionate use [9]**	Retrospective, noni-nterventional, non-comparative, single-center, observational study - **compassionate use**	The study included 8 categories of patients with neuroblastoma. Categories 1 and 2 (patients with primary refractory disease in bone and/or bone marrow only and patients with relapsed disease with incomplete response to salvage treatment in bone and bone marrow only, respectively) overlap with the subject population in Trials 12-230 and 201. Category 3 (patients with MRD, defined as patients with CR who were MRD positive by qPCR of bone marrow) provide supportive information on the efficacy evaluation of naxitamab. Patients from the remaining categories provide data for the evaluation of the safety of naxitamab.<br><br>Diagnosis of neuroblastoma as defined by international criteria (i.e., histopathology or bone marrow metastases plus high urine catecholamine levels).<br>Have been treated with naxitamab + GM-CSF under compassionate use between 01 June 2017 and 30 November 2018.	-	**NAX+GM-CSF**<br><br>All patients included in the application were treated after the same dosing regimen as in Trials 12-230 and 201: the dose level was 9.0 mg/kg per cycle, administered on Days 1, 3, and 5 of each cycle (3.0 mg/kg per infusion) in the presence of s.c. GM-CSF (Days –4 through 5).	**N=6+13**<br>The full analysis set (FAS) comprised the pooled Categories 1 and 2 encompassing 6 enrolments (6 unique patients). In addition, for Category 3, efficacy data on MRD was evaluated as supportive evidence for the effect of naxitamab. The Category 3 FAS comprised 13

Reference	Type of trial	Key inclusion criteria	Key exclusion criteria	Interventions (for patients with R/R disease)	Number of included patients
		Data were collected from patient medical records and recorded in eCRFs			enrolments (13 unique patients).

FAS – full analysis set; NAX- naxitamab, GM-CSF – granulocyte-macrophage colony stimulating factor.

Supplementary Table S12. Methodology of identified studies for dinutuximab beta in relapse/refractory neuroblastoma.

Reference	Type of trial	Key inclusion criteria	Key exclusion criteria	Interventions (for patients with R/R disease)	Number of included patients
**Wieczorek et al. 2023 [1]**	Retrospective study (review of the medical records), multicenter	Patients with HR-NBL (based on INSS) who received at least one cycle of DB combined with isotretinoin (±IL-2) as standard maintenance therapy in a first-line or the R/R setting. HR-NB was considered refractory if patients had received more chemotherapy cycles than permitted. HR-NBL was considered relapsed in patients whose disease had progressed (new lesions or the progression of existing lesions in patients without complete response (CR)) or relapsed (new lesions following CR) during or after first-line therapy and who received DB maintenance therapy in the second line or later.	-	**DB+isotretinoin±IL-2**<br><br>DB: continuous infusion of 10 mg/m^2/day on days 1–10 of each 35-day cycle, for up to five cycles.<br><br>Isotretinoin: 160 mg/m^2/day administered for 14 days for up to six cycles; the first cycle was administered before the first cycle of DB and on days 11–24 of each remaining cycle.<br><br>IL-2: in accordance with the prescribing information, IL-2 was only given in the R/R setting was administered at 3 $x10^6$ IU/m^2/day for 5 consecutive days in the week preceding the first cycle of DB and on days 2–6 of each remaining cycle	First line setting: N=37^<br>R/R setting: N=17
**APN311-304 [2]-[3]**	Prospective, open-label, single-arm Phase II trial	Patients aged 1–21 years with NB, according to the INSS criteria: primary refractory Stage 4 disease or had relapsed after primary Stage 4 disease, or had developed distant metastases following primarily localized NBL, and their tumour burden was controlled using conventional therapy but with measurable disease still present.	-	**DB**<br><br>DB: 10mg/m^2/day as continuous infusion over the first 10 days of each 35-day cycle for up to 5 cycles (in the absence of disease progression) without IL-2 or isotretinoin. DB was planned to be administered in the hospital setting in each cycle, but if well tolerated, it could be given in an outpatient setting from day 5 of cycle 1.	N=40 (N=38 included in efficacy analysis)
**Flaadt et al. 2023 [4]**	Prospective, open-label, single-arm Phase I/II trial, multicenter	Age 1-21 years at trial enrolment, relapsed/refractory INSS stage 4 NBL or relapsed MYCN-amplified stage 2-3 NBL, **and haplo-SCT as part of the relapse treatment**; protocol did not make recommendations on systemic chemotherapy and local treatment before haplo-SCT	-	**DB+IL-2 (low dose)**<br><br>DB: From 60 days after transplantation patients without GvHD were scheduled to receive DB as an 8-hour infusion of 20 mg/m^2 once per day on 5 consecutive days, for a total of 6 cycles given every 4 weeks.	N=68

Reference	Type of trial	Key inclusion criteria	Key exclusion criteria	Interventions (for patients with R/R disease)	Number of included patients
				IL-2: to avoid induction of GvHD, low-dose IL-2 was added SC only in cycles 4-6 on days 6, 8, 10 ($1x10^6$ IU/m^2).<br><br>Patients exhibiting complete response, partial response, or stable disease after cycle three received three more cycles. In case of response after cycle 6, patients were eligible to receive another three cycles.	
**Mueller et al. 2018 [6]/ APN311-303 [8]**	Open-label, single-arm, retrospective, single-center, compassionate use	Patients at ≥ 1 year and ≤ 45 years of age at treatment start with diagnosis of HN-NBL according to the INSS criteria, i.e. INSS stage 2, 3, 4, or 4s with MYCN amplification, or INSS stage 4 without MYCN amplification or relapsed or refractory NBL of any stage.<br>Off any standard or experimental treatments for at least 2 weeks prior to treatment start and fully recovered from the short term major toxic effects	Progressive disease, previous treatment with DB and/or ch14.19/SP2/0, if positive for HACA (human anti-chimeric antibody)	**DB+isotretinoin+IL-2**<br><br>DB: initially 50 mg/m^2 in their first treatment cycle in order to assess feasibility and tolerability of the treatment regimen. The majority of patients started with SC IL-2 in the first week, followed by a combination of DB and IL-2 in the second week. The total duration of a cycle varied between 28 and 35 days. In each cycle treatment ended with oral isotretinoin after the completion of the DB infusion. A total of up to 6 cycles was given.<br><br>IL-2: IL-2 was usually given SC at a dose of 6 x 10^6 IU/m^2/day. The majority of patient received it in two 5-day blocks (days 1-5 and 8-12). In these patients, IL-2 was given concurrently with DB on days 8-12. Initial patients, however, received IL-2 on days 1-5 only as they started with the combination of IL-2 and DB. Patients ≤ 12 kg were dosed according to body weight: $0.2x10^6$ IU/kg/day.<br><br>Isotretinoin: at a total daily dose of 160 mg/m^2/day administered in two equal oral doses twice a day for 14 days after the completion of the DB infusion. Doses were rounded to the nearest 10 mg. The starting day was either day 14 or day 21. Patients ≤12 kg were given 5.33 mg/kg/day divided into two equal doses given orally twice a day for 14 days.<br><br>In order to explore the safety and tolerability of the combination (DB+IL-2+isotretinoin) the first 4 patients were treated according to less intensive treatment schedule 1 in their first treatment cycle.	N=53 in reference [6], N=54 in reference [8]<br><br>Additional analysis: Patients (n= 29) with relapsed disease treated by LTI were matched with a control patient population (n= 27) from the AIEOP database (not treated with DB)

Reference	Type of trial	Key inclusion criteria	Key exclusion criteria	Interventions (for patients with R/R disease)	Number of included patients
**APN311-202 V1+V2 and V3 [5], [8]**	Phase I/II trial<br><br>**Stage I:** dose schedule finding phase (V1+V2) open-label, single-arm, multicenter	At study entry patients had to be > 1 year but ≤ 21 years of age.<br>Patients >21 years but ≤ 45 years of age, fulfilling the remaining criteria, could be enrolled in the study. These patients were to be analyzed separately and were not to be included in the dose finding schedule algorithm. The purpose for inclusion of the older patients was to enable the collection of tolerability data.<br>b) Patients had to be diagnosed with neuroblastoma according to the International Neuroblastoma Staging System (INSS) criteria.<br>c) Patients had to have received at least 1 previous high-dose treatment followed by stem cell rescue after conventional therapy.<br>d) Patients had to fulfill one of the following criteria:<br>• Patients with Stage 4 neuroblastoma either:<br>o on the current high-risk SIOPEN trial (HR-NBL-1/SIOPEN) either with primary refractory disease having had 2 or more than 2 front-line treatments or patients ineligible for the R2 randomization due to major delays after completed high-dose treatments, or<br>o standard high-risk front-line treatment (other than HR-NBL1(1.5)/SIOPEN) consisting of intensive induction, followed by high-dose treatment with stem cell rescue.<br>• Treated and responding relapse after primary Stage 4 disease, without signs of progression at study entry;<br>• Treated and responding disseminated neuroblastoma relapse having received autologous stem cell transplantation (ASCT) without signs of progression at study entry	Progressive disease, previous treatment with DB or ch14.19/SP2/0	**DB+isotretinoin+IL-2**<br><br>DB: applied from Day 8 to 18 of each 35-day cycle as a 10-day continuous infusion of 100 mg/m^2 per cycle.<br><br>IL-2: at the dose of $6x10^6$ IU/m²/day was given on Days 1-5 and Days 8-12 of each cycle.<br><br>Isotretinoin: applied for 14 days starting at cycle Day 22 at the dose of 160 mg/m^2/day.	N=44
	Phase II trial<br><br>**Stage II:** confirmatory phase to treat an expansion cohort (V3) – randomized controlled trial, parallel groups	Key inclusion criteria:<br>1. Were diagnosed with neuroblastoma according to the INSS criteria<br>2. Were aged > 1 year but ≥21 years of age at study entry (subjects >21 and ≤45 years of age could be enrolled and treated for the collection of tolerability data only)<br>3. Had received at least one previous high dose treatment followed by stem cell rescue after conventional therapy.<br>4. Fulfilled one of the following<br>• Stage 4 neuroblastoma and	Progressive disease, previous treatment with DB or ch14.19/SP2/0	Group I: **DB+isotretinoin**<br>DB LTI 5 cycles of 100 mg/m^2 DB-LTI (days 8-17) + 160 mg/m^2 oral isotretinoin (days 19-32)<br><br>vs<br>Group II: **DB+isotretinoin+IL-2**<br>DB LTI 5 cycles of 100 mg/m^2 DB-LTI (days 8-17) + 160 mg/m^2 oral isotretinoin (days 19-32) with IL-2 SC $6x10^6$IU/m^2 (days 1-5; 8-12)	Dinutuximab beta: N=81<br>Dinutuximab beta+IL-2: N=79

Reference	Type of trial	Key inclusion criteria	Key exclusion criteria	Interventions (for patients with R/R disease)	Number of included patients
		o on the current high-risk SIOPEN trial (HR-NBL-1(1.5)/SIOPEN) either with primary refractory disease having had two or more than two front-line treatments or subjects ineligible for the R2 or respectively R4 randomisation due to major delays after completed high-dose treatments<br>or<br>o standard high-risk front-line treatment (other than HR-NBL1(1.5)/SIOPEN) consisting of intensive induction, followed by high-dose treatment with stem cell rescue<br>• Treated and responding relapse after primary stage 4 neuroblastoma, without signs of progression at study entry;<br>• life expectancy ≥12 weeks.			
**Ladenstein et al. 2013 [7]**	Open-label, single-arm, multicenter, phase 1	Patients with biopsy-proven NBL (> 1 year) with refractory or recurrent disease were eligible. Patients after first-line therapy had to have evaluable disease. Following second-line chemotherapies, patients were **eligible without evidence of disease**; treatment had to be discontinued three weeks prior to study entry. **Patients with progression or previous antibody treatments were excluded.** Treatment with isotretinoin, growth factor or other immunomodulatory therapy needed to be completed at least 7 d before study entry.	-	**DB:** three dose levels (levels 1, 2 and 3 using 10, 20 and 30 mg/m^2/day respectively). One treatment cycle was planned. Patients were allowed to receive up to another two cycles provided they were progression-free; DB was administered daily as an eight-hour infusion over five consecutive days (days 0–4)<br><br>41 courses (10 × 3 courses, 5 × 2 courses, 1 × 1 course) were administered. Patients received DB courses of 10, 20 or 30 mg/m^2/ day, i.e., dose levels 1, 2 and 3 respectively, as an 8-hour infusion over 5 consecutive days. 16 completed the first course (level 1, 2 and 3 with 3, 10 and 3 patients each), 15 patients the second course (level 1, 2 and 3 with 3, 9 and 3 patients each) and 10 had a third course (level 1, 2 and 3 with 1, 6 and 3 patients each).	N=16

ASCR - autologous stem cell rescue; ASCT - autologous stem cell transplantation; DB – dinutuximab beta; GvHD – graft versus host disease; HR-NBL - high-risk neuroblastoma; INSS –International Neuroblastoma Staging System; IL-2 – interleukin 2; LTI – long term infusion; NBL – neuroblastoma, PO – per os (orally); R/R- relapse/refractory disease, SC – subcutaneous; SCT – stem cell transplantation; ^Due to the purpose of the review, only the results in the relapse/refractory subpopulation were described.

Supplementary Table S13. Assessment of prospective single-arm studies according to NICE criteria - part I.

Criterium	Study 12-201 (NCT03363373) [9]-[35]	Study 12-230 (NCT01757626) [9], [36]-[39]	Study 2PR01 – compassionate use [9]	Wieczorek et al. 2023 [1]	Mueller et al. 2018 [6]/APN311-303 [8]
**Case series collected in more than one center, i.e, multi-center study**	YES (1 point)	NO (0 points)	NO (0 points)	YES (1 point)	NO (0 points)

**Is the hypothesis/aim/objective of the study clearly described?**	YES (1 point)	YES (1 point)	YES (1 point)	YES (1 point)	YES (1 point)
**Are the inclusion/exclusion criteria (case definition) clearly reported?**	YES (1 point)	YES (1 point)	NO (0 points)	YES (1 point)	YES (1 point)
**Is there a clear definition of the outcomes reported?**	YES (1 point)	YES (1 point)	YES (1 point)	YES (1 point)	YES (1 point)
**Were data collected prospectively?**	YES (1 point)	YES (1 point)	NO (0 points)	NO (0 points)	NO (0 points)
**Is there an explicit statement that patients were recruited consecutively?**	NO (0 points)	NO (0 points)	NO (0 points)	NO (0 points)	NO (0 points)
**Were the main results/findings of the study clearly described?**	YES (1 point)	YES (1 point)	YES (1 point)	YES (1 point)	YES (1 point)
**Were the analyzed endpoints assessed in strata (groups of patients distinguished by, for example, the stage of disease advancement, abnormal test results, patient characteristics)??**	YES (1 point)	YES (1 point)	YES (1 point)	YES (1 point)	YES (1 point)
**Sum of points:**	**7 points**	**6 points**	**4 points**	**6 points**	**5 points**

Supplementary Table S14. Assessment of prospective single-arm studies according to NICE criteria - part II.

Criterium	APN311-304 [2]-[3]	Flaadt et al. 2023 [4]	APN311-202 Phase I [5], [8]	Ladenstein et al. 2013 [7]
**Case series collected in more than one center, i.e, multi-center study**	YES (1 point)	YES (1 point)	YES (1 point)	YES (1 point)
**Is the hypothesis/aim/objective of the study clearly described?**	YES (1 point)	YES (1 point)	YES (1 point)	YES (1 point)
**Are the inclusion/exclusion criteria (case definition) clearly reported?**	YES (1 point)	YES (1 point)	YES (1 point)	YES (1 point)
**Is there a clear definition of the outcomes reported?**	YES (1 point)	YES (1 point)	YES (1 point)	YES (1 point)
**Were data collected prospectively?**	YES (1 point)	YES (1 point)	YES (1 point)	YES (1 point)
**Is there an explicit statement that patients were recruited consecutively?**	NO (0 points)	NO (0 points)	NO (0 points)	NO (0 points)
**Were the main results/findings of the study clearly described?**	YES (1 point)	YES (1 point)	YES (1 point)	YES (1 point)
**Were the analyzed endpoints assessed in strata (groups of patients distinguished by, for example, the stage of disease advancement, abnormal test results, patient characteristics)??**	YES (1 point)	YES (1 point)	YES (1 point)	YES (1 point)
**Sum of points:**	**7 points**	**7 points**	**7 points**	**7 points**

Supplementary Table S15. Risk of Bias 2.0 assessment APN311-202 Phase II –RCT.

Study	D1 – Bias arising from the randomization process	D2 – Bias due to deviation from intended intervention	D3 –Bias due to missing outcome data	D4 – Bias in measurement of the outcome	D5 – Bias in selection of the reported results	Overall
**APN311-202 – randomized phase [5], [8]**	high	high	low	low	low	high

Supplementary Table S16. MAIC of OS: results.

	Sensitivity analysis #1 [A]	Sensitivity analysis #2 [B]	Sensitivity analysis #3 [C]	Sensitivity analysis #4 [D]	Sensitivity analysis #5 [E]	Sensitivity analysis #6 [F]
**Log-rank test, p value**	0.174	-	-	0.526	-	-
**HR (95% CI), p value**	1.84 (0.75 to 4.47), 0.181	2.16 (0.87 to 5.37), 0.096	1.52 (0.47 to 4.89), 0.480	1.41 (0.49 to 4.04), 0.527	1.36 (0.47 to 3.90), 0.572	1.35 (0.42 to 4.33), 0.615

[A] unadjusted comparison
[B] all patients from APN311-304 and APN311-202 trials (N=77); MAIC with adjusted variables: refractory, female, MYCN amplification, bone marrow only, bone and bone marrow
[C] with additional variables in MAIC: prior radiotherapy, Black race, % MYCN missing, % stage 3 INSS and % missing INSS
[D] unadjusted comparison; patients without IL-2 treatment in DB arm (N=29) vs naxitamab in Study 201
[E] MAIC; patients without IL-2 treatment in DB arm (N=29); adjusted variables: refractory, female, MYCN amplification, bone marrow only, bone and bone marrow
[F] MAIC; patients without IL-2 treatment in DB arm (N=29); adjusted variables: refractory, female, MYCN amplification, bone marrow only, bone and bone marrow, prior radiotherapy, Black
